# Monolithic Integrated Device of GaN Micro-LED with Graphene Transparent Electrode and Graphene Active-Matrix Driving Transistor

**DOI:** 10.3390/ma12030428

**Published:** 2019-01-30

**Authors:** Yafei Fu, Jie Sun, Zaifa Du, Weiling Guo, Chunli Yan, Fangzhu Xiong, Le Wang, Yibo Dong, Chen Xu, Jun Deng, Tailiang Guo, Qun Yan

**Affiliations:** 1 Key Laboratory of Optoelectronics Technology, College of Microelectronics, Beijing University of Technology, Beijing 100124, China; fuxixi321@163.com (Y.F.); 17801011216@163.com (Z.D.); fangzhuxiong@emails.bjut.edu.cn (F.X.); wangle316@emails.bjut.edu.cn (L.W.); donyibo@emails.bjut.edu.cn (Y.D.); xuchen58@bjut.edu.cn (C.X.); dengsu@bjut.edu.cn (J.D.); 2 National and Local United Engineering Laboratory of Flat Panel Display Technology, College of Physics and Information Engineering, Fuzhou University, Fuzhou 350116, China; gtl@fzu.edu.cn (T.G.); qunfyan@gmail.com (Q.Y.); 3 Department of Information and Automation, Library of Fuzhou University, Fuzhou 350116, China; yan_chunli@yahoo.com

**Keywords:** GaN micro-light-emitting diodes, two-dimensional materials, graphene, field effect transistors, monolithic integration

## Abstract

Micro-light-emitting diodes (micro-LEDs) are the key to next-generation display technology. However, since the driving circuits are typically composed of Si devices, numerous micro-LED pixels must be transferred from their GaN substrate to bond with the Si field-effect transistors (FETs). This process is called massive transfer, which is arguably the largest obstacle preventing the commercialization of micro-LEDs. We combined GaN devices with emerging graphene transistors and for the first-time designed, fabricated, and measured a monolithic integrated device composed of a GaN micro-LED and a graphene FET connected in series. The *p*-electrode of the micro-LED was connected to the source of the driving transistor. The FET was used to tune the work current in the micro-LED. Meanwhile, the transparent electrode of the micro-LED was also made of graphene. The operation of the device was demonstrated in room temperature conditions. This research opens the gateway to a new field where other two-dimensional (2D) materials can be used as FET channel materials to further improve transfer properties. The 2D materials can in principle be grown directly onto GaN, which is reproducible and scalable. Also, considering the outstanding properties and versatility of 2D materials, it is possible to envision fully transparent micro-LED displays with transfer-free active matrices (AM), alongside an efficient thermal management solution.

## 1. Introduction

With the rapid development of modern intelligent devices, the demand for power reduction and resolution enhancement of electronic displays has become higher and higher. Light-emitting diodes, or micro-LEDs, have a single-pixel area ≤ 2.5 × 10^−3^ mm^2^ (50 × 50 µm or less). They represent the development trend of the next generation of displays by virtue of their low power, weather fastness, and super-high resolution. Currently, micro-LEDs constitute a new research focus in major university labs and in the semiconductor industry. Nevertheless, as it is still being developed, the technology has not yet been commercialized. One of the major bottlenecks is the “massive transfer” [1]. Micro-LEDs are typically fabricated on gallium nitride (GaN)-based wafers, whereas the active matrix (AM) circuitry is made of Si-based complementary metal-oxide-semiconductor (CMOS) devices. A massive transfer of hundreds of thousands of micro-LED pixels is needed to bond the micro-LEDs with their individual AM driving elements and this is a huge technical obstacle, since the process relies critically on alignment accuracy, bonding strength, and reliability. It is expected that the alignment accuracy should be within ± 0.5 µm, and the yield higher than 99.9999%. Currently, most researchers are focusing on developing advanced transfer technologies. However, we believe that another route that is very promising and should receive more attention is the monolithic integration of micro-LEDs and their driving circuits. This route can bypass the massive transfer, reduce cost and technological difficulties, and be more reliable in applications. Previously, GaN-based micro LEDs were integrated with GaN high-electron-mobility transistors (HEMTs) [2] or metal-oxide-semiconductor field-effect transistors (MOSFETs) [3]. GaN transistors generally have high breakdown voltages [4,5] and high working frequencies [6,7]. However, because of the lattice mismatch, it is hard to grow high-quality GaN LEDs and transistors directly on top of each other. Furthermore, the deposition temperatures of the two are quite different, making the growth processes of the two materials not compatible [2,3].

Graphene, a monolayer of graphite, is known as a new wonder material. It integrates many outstanding properties into one material (e.g., high carrier mobility, minimal thickness, high optical transmittance, high thermal conductivity, excellent chemical stability, mechanical strength, and flexibility) and hence, has a great potential in nanoelectronics [8]. It can be used as the channel material for transparent high-frequency transistors [9]. Furthermore, it can be used in transparent electrodes for LEDs [10]. Compared with traditional indium tin oxide (ITO), graphene electrodes have a broader spectrum and are more mechanically flexible and chemically stable. Graphene has been used in GaN LEDs and has shown potential for solving the current crowding problem caused by the resistive *p*-GaN capping layer of LEDs [11,12].

In this paper, we have designed and fabricated GaN micro-LEDs with graphene transparent electrodes and graphene field-effect driving transistors (GFET). To the best of our knowledge, this is the first proof-of-principle study on the monolithic integration of GaN micro-LEDs with their AM driving GFET. The role of graphene is two-fold: it acts as a transparent conducting film for the micro-LEDs and also as the channel material for the driving FETs to control the micro-LEDs. We demonstrate that graphene grown by chemical vapor deposition (CVD) can effectively spread the current in the *p*-GaN, making the lighting more uniform, while the field effect in the graphene can be used to tune the current in the integrated device. This technique is intrinsically scalable and compatible with the semiconductor industry. Although the present study uses graphene grown ex situ on a Cu foil, the method can be extended to graphene grown in situ on GaN [13]. Also, channel material may not be limited to graphene. Considering recent advances in large-scale growth of other two-dimensional (2D) materials, e.g., MoS_2_ with higher transistor on–off ratios [14], this research can be viewed as pioneering work which reveals the promising future of combining emerging 2D materials with traditional bulk semiconductors to resolve the issue of massive transfer that exists currently in the micro-LED community.

## 2. Experimental Procedures

**Figure 1:**
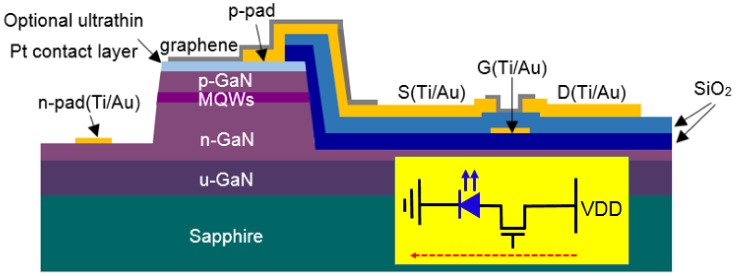
Schematic diagram of the monolithic integrated micro-light-emitting diodes (micro-LED)/graphene field-effect driving transistors (GFET) device. Some semiconductor layers are omitted for simplicity. The inset is its equivalent circuit, where the micro-LED is connected in series with the driving transistor (AM). The red arrow indicates the direction of the current flow. This figure is not drawn to scale. GaN, V_DD_, G, D, S and MQW denote gallium nitride, total applied voltage of the integrated device, gate, drain, source and multiple quantum well, respectively.

Figure 1 is a schematic illustration of the structure of the integrated GaN micro-LED and GFET device, which was fabricated on a commercial GaN LED epitaxial wafer with c-face-patterned sapphire substrate (PSS). The epi-layers were grown by metalorganic chemical vapor deposition (MOCVD) using a Veeco K465i system. From bottom to top, on the 17 nm buffer layer, there were in turn 2.5 μm *n*-GaN, 74 nm *n*-AlGaN, 131.75 nm multiple quantum well (MQW, InGaN/GaN), 40.62 nm electron-blocking layer (six periods of AlGaN/GaN = 3.61 nm/3.16 nm superlattice), and 92 nm *p*-GaN. The device fabrication process flow was as follows. First, mesa areas of the GaN micro-LEDs were defined by lift-off photolithography carried out in 2 nm Pt with 110 nm Ni sputtered onto the GaN LED wafer. The Pt/Ni layer was on the GaN mesa top, where Pt was used to improve the electrical contact between the transparent electrode and *p*-GaN, and Ni was used as the hard mask in the dry-etching. Inductively coupled plasma (ICP) etching was used to etch the semiconductor down to the *n*-GaN (1.25 μm deep) using a SiH_4_/Cl_2_ gas mixture. The Ni mask was then wet-etched away. At 300 °C, plasma-enhanced CVD (PECVD) was used to grow 300 nm SiO_2_ onto the sample as an insulation layer between the micro-LED and the graphene transistor. The patterning of this SiO_2_ layer was achieved by using buffered oxide etch (BOE). The buried back gate of the transistor was fabricated by another lift-off lithography with a sputtered 15 nm Ti/100 nm Au metal layer, as shown in Figure 1. PECVD was used for a second time to grow 300 nm SiO_2_, followed by BOE patterning to bury the gate. This layer of silicon dioxide served as the gate dielectric. Afterwards, a third lift-off lithography was used to pattern the sputtered 15 nm Ti/130 nm Au in order to form the *p*, *n* metal electrodes of the micro-LED, as well as the source and drain of the GFET. The *p*-pad of the micro-LED was connected to the source of the transistor. Finally, the graphene was grown by standard CVD on Cu foil using CH_4_ as the precursor (Shenzhen Jingge Nano Technology, Shenzhen, China). After the graphene CVD, the Cu foil was etched off, and the graphene transferred to the GaN wafer using standard graphene transfer technology [15]. The graphene, patterned by photolithography and oxygen plasma etching, functioned as the transparent electrode on the micro-LED and the channel in the driving FET.

The inset of Figure 1 is the equivalent circuit of the integrated micro-LED/GFET device. The micro-LED was essentially a GaN *pn* junction inserted with multiple quantum wells. When the injected current was large enough, spontaneous emission of photons was initiated by the recombination of electron–hole pairs. The GFET was connected with the micro-LED in series. Its gate capacitively coupled to the graphene channel. The Fermi energy in the channel was tuned by controlling the gate voltage, which was then translated into the modulation of the channel current *I*_d_. In this way, the current flowing into the micro LED and thus the electroluminescence could be adjusted.

## 3. Results and Discussion

**Figure 2:**
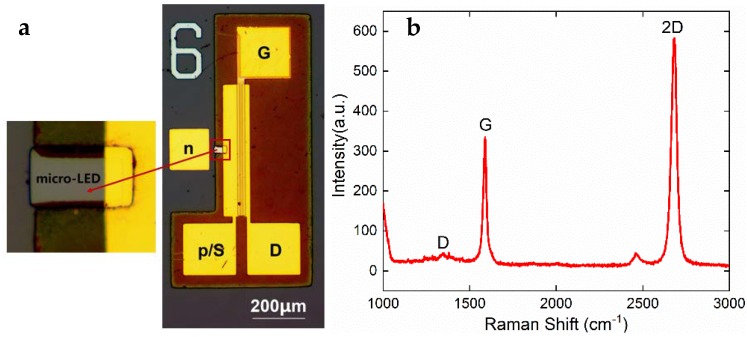
(**a**) Optical microscopy image of the micro-LED/GFET-integrated device. The mesa of the GaN micro LED is magnified; (**b**) Raman spectrum of the graphene monolayer.

Figure 2a shows an optical micrograph of the micro-LED/GFET device. The mesa area of the micro LED was 30 μm × 50 μm, and the graphene channel of the driving transistor was 12 μm × 480 μm. The *p* electrode of the micro-LED was connected to the source pad of the transistor. The S, D metal pads of the GFET were sitting on top of the double-layer SiO_2_ film. It can be seen that the G pad was a little blurred, because the gate was buried in between the two SiO_2_ layers, as shown in Figure 1. Figure 2b is a typical Raman spectrum measured in graphene. The G band and 2D band peaks at ∼1600 cm^−1^ and ∼2690 cm^−1^ are signatures of *sp*^2^ hybridized graphitic carbon. The *I*_G_/*I*_2D_ ratio was approximately 1/2, and the full width at half maximum (FWHM) of the 2D peak was ∼37.92 cm^−1^, indicating the graphene was a monolayer [16]. The D peak is exceedingly small, meaning that there were few disorders in the graphene lattice; therefore, the quality of the as-grown graphene was satisfactory. 

**Figure 3:**
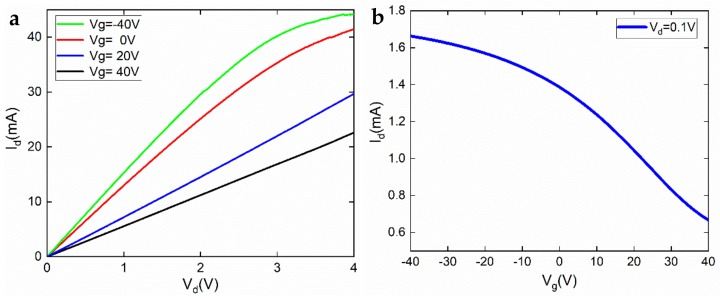
(**a**) Output and (**b**) transfer properties of the GFET measured at room temperature.

First, we measured discrete components’ performances. The static (DC) properties of the GFET were characterized at room temperature without special treatment, e.g., vacuum annealing. Figure 3a,b shows the output and transfer properties, respectively. In the GFET, 0–4 V (*V*_d_) was swept between the source and drain, while the gate voltage *V*_g_ was altered in steps (−40 V, 0 V, 20 V, and 40 V). In Figure 3a, one can see that with increasing gate voltage, the slope of the output curves got smaller, indicating an increasing channel resistance. Clearly, the channel was *p*-type. When *V*_d_ was fixed at 0.1 V and the gate voltage *V*_g_ was swept between −40 V and 40 V, the transfer curve shown in Figure 3b could be obtained. The Dirac point was at *V*_g_ > 40 V, which means the graphene was far from intrinsic (heavy *p*-doping). This can primarily be ascribed to the photolithography procedure, where the photoresist residue is known to dope the graphene severely [17]. Unfortunately, this doping significantly damages graphene mobility. Apart from the doping effect introduced during the device processing, direct exposure to open air without passivation leads to adsorption of H_2_O, which also reduces the carrier mobility. Using a formula based on a capacitor model:

(1) $$\mu =\frac{\delta I_{d}}{C_{g}V_{d}\delta V_{g}}\frac{L}{W}$$

where *μ*, *C*_g_, *L*, *W* are the carrier mobility, gate capacitance, gate length, and gate width, respectively, the hole mobility in the GFET was calculated to be 696 cm^2^·V^−1^·s^−1^ (the value $\frac{\delta I_{d}}{\delta V_{g}}$ was taken when *V*_g_ = 25 V). We note that this is the field-effect mobility, and the calculation depends on many device parameters, e.g., interface trap density. Furthermore, we used a simplified model without considering the quantum capacitance. The standard Hall measurement gave a more accurate estimation of graphene mobility on polyethylene terephthalate (PET) substrate, showing 1042 cm^2^·V^−1^·s^−1^. The transconductance *g*_m_ of the GFET could be directly calculated from Figure 3b. At *V*_g_ = 25 V, the transconductance reached its maximum, which was about 0.067 mS/mm (normalized to the gate width). The sheet resistance of the monolayer CVD graphene was approximately 4 kΩ/□.

Graphene is a semimetal with no bandgap. Thus, graphene–metal contacts are generally very good. The specific contact resistivity for typical graphene–metal contacts is 10^−5^ or 10^−6^ Ωcm^2^ [18], with some of the best examples being 10^−7^ Ωcm^2^ or lower [19]. For our graphene, the typical value is ρ_c_ = 1 × 10^−6^ Ωcm^2^ [20]. The total overlapping area in the integrated device was 480 µm × 55 µm, which means the total contact resistances arising from the graphene–metal contacts added up to less than 0.01 Ω. Clearly, this figure can be ignored. Good graphene–metal contact behaviour was also confirmed by another work of ours [11].

**Figure 4:**
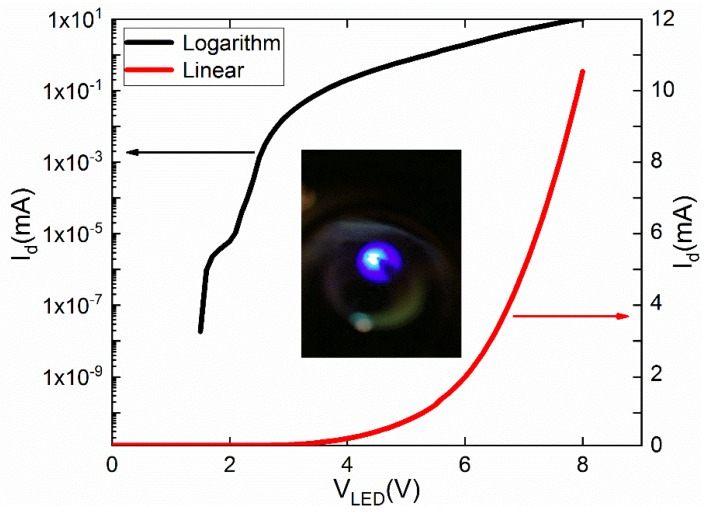
Current–voltage characteristics of the micro-LED plotted in linear and logarithmic scales. The inset is an electroluminescence photo of the device measured in a probe station.

Figure 4 plots the I–V properties of the GaN micro-LED. The black and red curves are the logarithmic and linear plots, respectively. In this figure, the turn-on voltage is shown to be around 5.8 V, a higher voltage than that of commercial GaN LED devices. This was of course due to our fabrication technique but, more importantly, to the fact that the graphene’s Fermi level did not match that of *p*-GaN. The forward differential resistance (series resistance) of the micro-LED was estimated to be as large as 190 Ω, which was also due to the nonoptimal graphene–GaN contact. The 2 nm Pt layer can bridge the Fermi levels of the graphene and *p*-GaN, improving the Ohmic contact to some extent. Currently, there is no stable and mature doping technology for graphene. In the future, if effective and stable doping can be realized, the graphene work function could be greatly improved, and the turn-on voltage would ultimately be reduced. At 8 V forward voltage *V*_LED_, the work current *I*_d_ was about 10.5 mA, which is large enough for a micro-LED. The insert of Figure 4 is a photograph taken when the micro-LED was turned on. It is an intuitive image demonstrating the uniformity and high intensity of the lighting. Because of the much higher conductivity of graphene compared with *p*-GaN, the graphene transparent electrode helped spread the current effectively along the *p*-GaN mesa surface, solving the current crowding problem. The high transmittance of graphene ensured the light was effectively emitted to the external world. 

**Figure 5:**
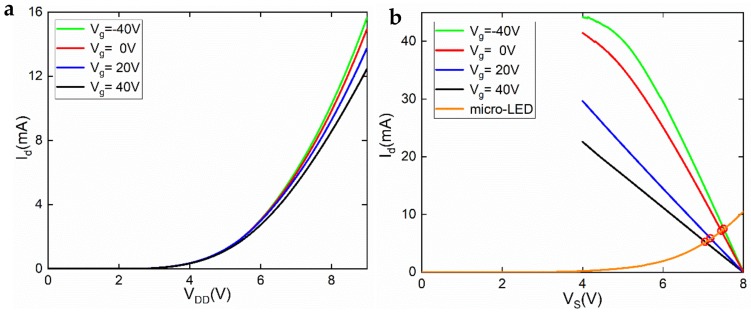
(**a**) The overall I–V curve of the integrated micro-LED/GFET device; (**b**) demonstration of the static working mechanism of the integrated device. The device current is plotted against *V*_s_, with *V*_DD_ fixed at 8 V. The crossing points are referred to as static work points in this paper.

Finally, we examined the properties of the GaN micro-LED/GFET as an integrated device. *V*_DD_ of 0–9 V was swept on the drain electrode of the GFET, with the *n* electrode of the micro-LED grounded, as shown in the inset of Figure 1. The I–V properties are plotted in Figure 5a, where the gate voltage *V*_g_ is shown at −40 V, 0 V, 20 V, and 40 V. In this figure, while tuning *V*_g_, the current *I*_d_ showed some changes when the *V*_DD_ was relatively large. The current reached its maximum when *V*_g_ was −40 V and reached its minimum at *V*_g_ = 40 V. When *V*_DD_ was 9 V, the difference between *I*_max_ and *I*_min_ was approximately 3.5 mA. This confirmed that the GFET can indeed be used to control the micro-LED in an AM fashion. Figure 5b plots the I–V curves of the micro-LED and its graphene driving transistor in the same figure and can be used to determine the static working point (the crossover points in this figure) of the integrated device. In this figure, *V*_DD_ was set to be 8 V,(maximum voltage on the horizontal axis). The device current *I*_d_ (work current of both the micro-LED and the GFET) was plotted against the voltage on the transistor source, *V*_s_, which is also the voltage on the *p* pad of the micro-LED. Clearly, when *V*_g_ was tuned from negative to positive, the voltage of the crossover point, *V*_s_, was reduced, and the current *I*_d_ was reduced correspondingly. This is because the graphene channel resistance increases when the gate voltage becomes more positive, due to the hole conduction mechanism. The voltage drop across the GFET (*V*_DD_–*V*_s_) in the series circuit will therefore increase, leading to a reduction of voltage on the micro-LED. As the I–V relation of the micro-LED is exponential, a small decrease in the voltage on the *p* electrode, *V*_s_, will result in a big decrease in the work current *I*_d_. This notwithstanding, compared to micro-LEDs driven by MOSFETs [3], the current tunability in our device was modest. At the gate voltage range shown in Figure 5, the micro-LED could not be totally turned off (*V*_s_ cannot be < 5.8 V). This can be attributed to the process-deteriorated graphene quality (mainly mobility). Furthermore, graphene is gapless, which means the GFET has a low on–off ratio. Also, the buried gate was very far from the channel, and the gate dielectric was not high-k material (300 nm SiO_2_). In the future, the channel material of the AM driving transistors can consist of other emerging 2D materials that have energy bandgaps, such as MoS_2_ and other transition-metal dichalcogenides (TMDs). With the technology of 2D materials becoming more and more developed, mobility, and consequently the transconductance of the FET channel, can be much improved. The size and aspect ratio of the gate can be optimized, and the gate dielectric can be replaced by high-k insulators [21] to further boost transconductance.

The integrated devices reported in this paper and in reference [3] can both bypass the technical obstacles of massive transfer, accurate alignment, and bonding of micro-LED pixels. This bypass is a great advantage when compared with devices with a traditional MOSFET driving module fabricated on a separate Si wafer. When comparing our work with that described in reference [3], the performances of the micro-LEDs are very similar. The final integrated devices also work at similar voltage and current ranges. However, our GFET transconductance is four orders of magnitude lower than that of GaN MOSFETs [3], resulting in a much weaker tunability. On the other hand, unlike GaN MOSFETs, which have been researched for decades, 2D material FETs are still in their infancy, and there is still a lot of room to improve, as was discussed earlier. Most importantly, our device concept and fabrication technology are much simpler than those in reference [3], where two GaN dry-etching steps of different depths are required to fabricate the micro-LED and the MOSFET, respectively.

## 4. Conclusions

In this paper, we have designed, fabricated, and characterized a monolithic integrated device composed of a GaN micro-LED and a driving element, namely, a graphene transistor. Meanwhile, the graphene on the GaN mesa served as the transparent electrode for the micro-LED. This is the first demonstration of combining GaN micro-LEDs with AM GFETs to bypass the technical difficulty of massive transfer, which exists currently in the micro-LED community. By tuning the gate voltage of the GFET, the micro-LED current and the light intensity could be adjusted. The performance of the integrated device needs further improvement. With research booming on 2D materials other than graphene, we believe this research opens the gateway for combining traditional bulk semiconductors with ultrathin 2D materials in a bid to improve micro-LED applications. The 2D materials can be grown in situ onto GaN to achieve better process reproducibility. Furthermore, since 2D materials such as graphene often have very high thermal conductivity, the heat spreading effect should not be overlooked. This is especially important for micro-LED displays, where the pixel density is super-high, and an appropriate thermal management solution is a must. The thermal aspect of the monolithic integrated device is a subject for future work. Additionally, as 2D materials are atomically thin and transparent, as long as the metal electrodes are replaced by transparent conductors, e.g., ITO, the whole display can be made transparent, which is well in line with the future trend of development.
